# Entomo-Virological *Aedes aegypti* Surveillance Applied for Prediction of Dengue Transmission: A Spatio-Temporal Modeling Study

**DOI:** 10.3390/pathogens12010004

**Published:** 2022-12-20

**Authors:** André de Souza Leandro, Mario J. C. Ayala, Renata Defante Lopes, Caroline Amaral Martins, Rafael Maciel-de-Freitas, Daniel A. M. Villela

**Affiliations:** 1 Centro de Controle de Zoonoses, Secretaria Municipal de Saúde, Foz do Iguaçu 85869-657, PR, Brazil; 2 Laboratório de Mosquitos Transmissores de Hematozoários, Instituto Oswaldo Cruz, Fiocruz, Rio de Janeiro 21040-360, RJ, Brazil; 3 Programa de Computação Científica, Fundação Oswaldo Cruz, Rio de Janeiro 21040-900, RJ, Brazil; 4 Escuela de Optimización, Infraestructura y Automatización, Politécnico Grancolombiano, Bogotá 110231, Colombia; 5 Programa de Pós-Graduação em Políticas Públicas e Desenvolvimento, Universidade da Integração Latino Americana—UNILA, Foz do Iguaçu 85866-000, PR, Brazil; 6 Department of Arbovirology, Bernhard Nocht Institute of Tropical Medicine, 20359 Hamburg, Germany

**Keywords:** dengue, Zika, chikungunya, entomological surveillance, vector control, vectorial capacity, disease transmission, epidemiology, arbovirus

## Abstract

Currently, DENV transmitted primarily by *Aedes aegypti* affects approximately one in three people annually. The spatio-temporal heterogeneity of vector infestation and the intensity of arbovirus transmission require surveillance capable of predicting an outbreak. In this work, we used data from 4 years of reported dengue cases and entomological indicators of adult *Aedes* collected from approximately 3500 traps installed in the city of Foz do Iguaçu, Brazil, to evaluate the spatial and temporal association between vector infestation and the occurrence of dengue cases. Entomological (TPI, ADI and MII) and entomo-virological (EVI) indexes were generated with the goal to provide local health managers with a transmission risk stratification that allows targeting areas for vector control activities. We observed a dynamic pattern in the evaluation; however, it was a low spatio-temporal correlation of *Ae. aegypti* and incidence of dengue. Independent temporal and spatial effects capture a significant portion of the signal given by human arbovirus cases. The entomo-virological index (EVI) significantly signaled risk in a few areas, whereas entomological indexes were not effective in providing dengue risk alert. Investigating the variation of biotic and abiotic factors between areas with and without correlation should provide more information about the local epidemiology of dengue.

## 1. Introduction

The Flaviviridae and Togaviridae families are among the most important public health arboviruses listed in the International Catalog of viruses, including the four dengue virus serotypes (DENV1, DENV2, DENV3 and DENV4), Zika virus (ZIKV), yellow fever (YFV), West Nile virus (WNV) and chikungunya virus (CHIKV) [1]. Dengue is an acute febrile disease, ranging from asymptomatic to severe and fatal, transmitted by *Aedes* mosquitoes. The incidence of dengue fever has increased 30-fold worldwide over the last five decades, and its geographic distribution has increased as well. Therefore, DENV nowadays affects approximately one in three people annually in populated areas of tropical and subtropical regions of the planet. Infected humans, regardless of their clinical condition, are reservoirs and amplifiers of arbovirus and, therefore, a source of infection for vectors [2,3,4].

*Aedes aegypti* is the primary vector of dengue in most parts of the world, although at a lower extent *Aedes albopictus* and *Aedes polynesiensis* have been implicated in some outbreaks. The prominent role of *Ae. aegypti* relies on its close association with humans. Females preferentially bite humans rather than other vertebrates and lay eggs in man-made containers in the surroundings of human dwellings, generally breeding sites not fully covered with few organic materials for larval feeding. Clearly, uncontrolled urbanization, increasing temperatures and lack of effective sustainable vector control tools boosted *Ae. aegypti* adaptation to urban environments with global distribution [5,6,7,8,9].

Since there is no drug or universal vaccine to mitigate dengue transmission, the maintenance of *Ae. aegypti* population density below a critical threshold is still the most recommended way to control arboviruses [10]. However, traditional approaches have shown limited success in targeting the mosquito population and, consequently, reducing dengue transmission. Briefly, mechanical control (targeting breeding sites) is laborious and requires military discipline over the long-term to be effective and an unaffordable team of health agents to visit houses fortnightly [11,12,13]. Chemical control (insecticide application) is jeopardized by the dissemination of alleles conferring insecticide resistance to different classes of compounds [14,15]. Biological control is environmentally friendly, but production and distribution of the adopted species has proven to be challenging. Furthermore, endemic countries in general do not have a timely routine for arbovirus surveillance to guide local decision makers, i.e., the precariousness of the current surveillance and vector control tools often contribute to the failure of maintaining a reduced *Ae. aegypti* population size [11,16,17].

In several tropical endemic countries, entomological surveillance relies on indicators based on larval sampling. However, there are numerous reports available showing those larval-sampling indicators have lower correlation with adult density and, thus, are unable to identify the hotspots of disease transmission as well as predict the arboviruses transmission risk [18,19]. In turn, the addition of traps for sampling the adult population of *Ae. aegypti* can provide entomological indicators based on adult sampling [20]. Those indexes have shown an improved ability of predicting the occurrence of DENV outbreaks after four weeks of trap inspection [21].

There is an urgent need to evolve surveillance practices aimed at the control of diseases caused by arboviruses to avoid outbreaks and epidemics and their consequences. The heterogeneity and spatio-temporal dynamism of vector infestation and intensity of arbovirus transmission make it necessary to reorganize surveillance aimed at vector and arbovirus control based on risk evidence and constantly evaluate it in real time. Effective arbovirus surveillance must be able to accurately predict the timing and location of an outbreak.

In this work, we evaluated the spatial association between entomological indicators based on adult *Ae. aegypti* female sampling and entomo-virological indicators with dengue cases in the 73 areas of Foz do Iguaçu city, Southern Brazil, with data from January 2017 to December 2020. The ultimate goal is to provide local health managers with a stratification of transmission risk, so vector control activities can be directed toward those areas rather than uniformly conducted over the full city extension.

## 2. Methods

### 2.1. Study Site

This study was conducted in the city of Foz do Iguaçu (25°30′58″ S, 54°35′07″ W), Paraná State, Brazil. It is located on an epidemiologically critical area of the country since there exists a triple border with Argentina and Paraguay, with intense mobility across the countries’ border cities. Foz do Iguaçu has an estimated population of 260,000 people and is divided into 73 urban areas of ≈1500 premises each. Three rural areas were not included in this study. The geographical units called areas are territories where health agents from the municipality of Foz do Iguaçu work are sectorized. This area integrates the territorial base of primary health care professionals and endemic disease control teams. Each area has an average of 1500 properties and approximately 3500 inhabitants. The climate is characterized by hot and humid summers (average above 27 °C) and cold to mild winters (mean below 15 °C), with annual rainfall of 1850 mm.

### 2.2. Adult Mosquito Collection

The city of Foz do Iguaçu has a total of 3476 Adultraps (Berdon, https://adultrap.com.br, accessed on 17 December 2022) installed over the city since January 2017. As a rule, one trap is found in the peridomestic environment for every 25 premises. The Adultrap is originally designed to capture gravid *Ae. aegypti* female mosquitoes during oviposition since it uses water as the principal attractant. Water remains confined in a compartment at the bottom of the trap that the mosquitoes cannot access, thus deterring egg laying [22]. As Adultraps do not become breeding grounds, the traps remain active with water, permanently installed on the properties. Health agents visit all 3476 Adultraps every 2 months, when agents concomitantly conduct larval surveys to adhere with the Brazilian Ministry of Health guidelines. Therefore, the periodicity of trap inspection was a balance made by Foz do Iguaçu public health decision makers that considered factors such as the number of traps in the city (3476), the personnel availability for trap sampling (80–100 health agents), resources for molecular screening and federal government (Brazilian MoH) guidelines. This study used data gathered from January 2017 to December 2020. Therefore, the 3476 Adultraps were inspected 24 times in the same premises, a total of 83,424 trap inspections.

### 2.3. Entomological Indexes

For this work, we continued evaluating the three indicators based on sampling adult *Ae. aegypti* that showed ability to forecast dengue outbreaks in Foz do Iguaçu [23]. The trap positivity index (TPI) is the number of positive traps among the total number of traps inspected, multiplied by 100; the adult density index (ADI) is the total number of *Ae. aegypti* mosquitoes captured divided by the total number of inspected traps, multiplied by 100; and the mosquitoes per inhabitant index (MII) is the total number of adult *Ae. aegypti* mosquitoes collected, divided by the number of persons in each house with an Adultrap, multiplied by 1000. All indexes were estimated for every 2 months during 2017–2020, i.e., a total of 24 observations per index. Since entomological indicators based on larval surveys such as the house index (HI) and Breteau index (BI) had a low performance to predict arbovirus outbreak in the context observed in Foz do Iguaçu, we decided to keep it out of the current analysis [23].

### 2.4. Entomo-Virological Index

All mosquitoes caught in the Adultraps were collected, but those alive at the time of trap inspection were placed in vials separate from the dead ones. Samples were sent to the entomology laboratory, where taxonomic identification using appropriate keys were carried out. Those live mosquitoes identified as female *Ae. aegypti* were separated from the other mosquitoes and placed in cryogenic tubes for further diagnosis of arbovirus infection by quantitative real-time PCR (qPCR), whereas those captured dead were identified, recorded and discarded. The entomo-virological screening provided the entomo-virological index (EVI) [23]. We calculated the EVI as the number of positive specimens for ZIKV, CHIKV and DENV serotypes in the city block at the epidemiological week.

### 2.5. RNA Extraction and Real-Time qPCR

We extracted viral RNA from *Ae. aegypti* mosquitoes by using the MagMAX Viral/Pathogen Nucleic Acid Ultra Isolation KIT, according to the manufacturer’s instructions. As described above, electromagnetic mixing beads were added to 1.5 mL tubes with single or pooled mosquitoes and macerated by using TissueLyser II (QIAGEN Shenzhen Company Limited 6 e 7F, R3-B, High-Tech Industrial Park, Nanshan District, Shenzhen, China, 518057). After RNA extraction, we separated an aliquot of 2 µL from each sample and used this to read the concentration of viral RNA recovered in a NanoDrop OneC Spectrophotometer (Thermo Fisher Scientific 3411 Silverside Road Tatnall Building, Suite 100, Wilmington, DE 19810, U.S.A). For arboviral genome amplification, we used the ZDC Biomol Kit (Rua Professor Algacyr Munhoz Mader, 3775-Cidade Industrial de Curitiba, Curitiba-PR, 81350-010), which enables identification of ZIKV, CHIKV and differentiation of DENV serotypes with an internal control (IC) of the reaction that uses probes specific to each molecular target. We used a 96-well QuantStudio 7 Flex Real-Time PCR System (Thermo Fisher Scientific 3411, Silverside Road, Tatnall Building, Suite 100, Wilmington, DE 19810, USA) for PCR and analyzed results by using Quant Studio Design and Analysis Software versions 1.3.1 and 1.5.1. We considered samples positive when the amplification plot curve exceeded the specific threshold for each target.

### 2.6. Data Analysis

This work has the main purpose of evaluating whether the adult-base indicators would alert to a posterior increase in dengue cases in the 73 urban areas of Foz do Iguaçu. We evaluated this hypothesis by comparing the number of dengue cases per epidemiological week to four indicators (TPI, ADI, MII and EVI) during the same epidemiological week and in 1, 2, 3 and 4 weeks before. Remarkably, TPI, ADI and MII were based on adult sampling in Adultraps, whereas EVI refers to collecting naturally infected *Ae. aegypti* mosquitoes. The study database contains 15,184 individual records of the number of dengue cases, adding the values of TPI, ADI, MII and EVI per area per epidemiological week. This database listed the variables in 73 areas of Foz do Iguaçu per 208 epidemiological weeks. Each record has the number of dengue cases, and the other variables only have values in 1714 records, because the infestation indexes and EVI were calculated from periodic samples. We implemented a Bayesian model for representing the dengue cases using a spatial random effect, a temporal random effect and a spatial random effect associated to each co-variable. We labelled spatial variation as $i=\left\{1,2,\ldots ,73\right\}$ (73 areas), and we labelled the temporal variation as $t=\left\{1,2,\ldots ,208\right\}\;$ of 208 weeks from January of 2017 to December of 2020. $y_{it}$ represents the number of dengue cases in the area *i* at *t* week, and we modelled it using a Poisson distribution with mean $\lambda_{it}$:

(1) $$y_{it}=Poisson\left(\lambda_{it}\right)$$

where $\lambda_{it}=\rho_{it}\epsilon_{it}$ with $\rho_{it}$ as incidence rate and $\epsilon_{it}$ as offset. Population by area was used as offset. The rate $\rho_{it}$ was described by a linear predictor in the logarithmic scale:

(2) $$\nu_{it}=\log\left(\rho_{it}\right)=\alpha +\gamma_{i}+\delta_{t}+\sum_{k=1}^{4}\beta_{ki}\times X_{kit}$$

where α represents the average dengue incidence in all areas, $\gamma_{i}$ represents the spatial random effect according to Besag–York–Mollié (bym) model, $\delta_{t}$ represents the temporal random effect according to random walk model of order one (rw1) and $\beta_{ki}$ represents the spatial variable-dependent random effect in the area *i* interacting with the variable $X_{kit}$ with $k=\left\{1,\;2,\;3,\;4\right\}$ (one index per type: TPI, ADI, MII and EVI) at time *t* according to independent random effects (iid). We apply five variations of this model: variables at epidemiological week *t* ($X_{kit}$), and variables with lags from 1 to 4 weeks: $X_{ki\left(t-1\right),}\;X_{ki\left(t-2\right)},\;X_{ki\left(t-3\right)},\;X_{ki\left(t-4\right)}$. We estimate the spatial, temporal and variable-dependent effects applying this model with the R-INLA tool. Therefore, this implementation provides how much the number of dengue cases are explained by the temporal effect, independent spatial effect and, finally, the variable-dependent effects. In particular, the tool provides the distribution of parameters $\beta_{ki}$ associated with indexes. These effects are additive in the logarithmic scale and, hence, multiplicative for the incidence. Finally, the posterior probabilities signal if these multiplicative effects increase the incidence above the levels from the estimated total of the city level plus temporal and independent spatial effects. For instance, values > 0.8 can be considered high probabilities, although there are uncertainty intervals for these variables. We also assess the credibility intervals to find if these effects are significant.

## 3. Results

**Figure 1:**
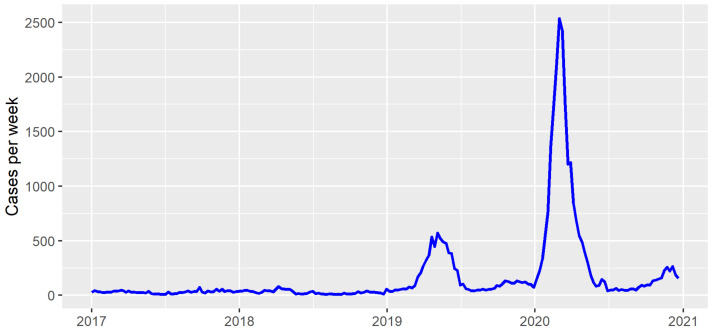
Dengue cases per week in Foz do Iguaçu from 2017 to 2021.

**Figure 2:**
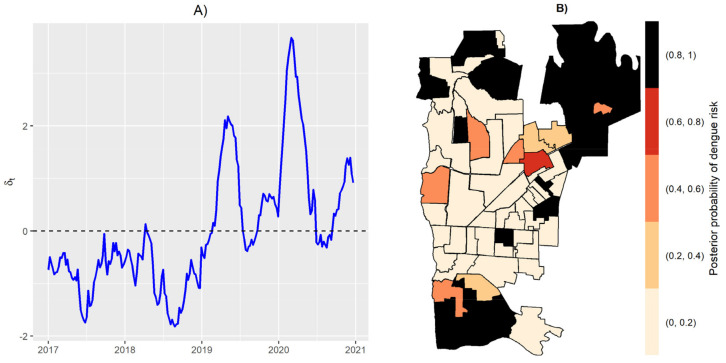
Temporal random effect (**A**), and posterior probability of dengue risk from spatial random effect (**B**).

The incidence of dengue in Foz do Iguaçu, Brazil, varied over the years from 2017 to 2020. Low incidences were observed in years 2017 and 2018, whereas separate outbreaks were observed in years 2019 and 2020 (Figure 1). As expected, the temporal effect reached high values in those peaks of 2019 and 2020 (Figure 2A). The independent spatial effect indicated several areas with greater risk of dengue in the city. The temporal effect and independent spatial effect capture much of the variation in the dengue cases. Still, some variation could be explained by the index-dependent parameters.

**Figure 3:**
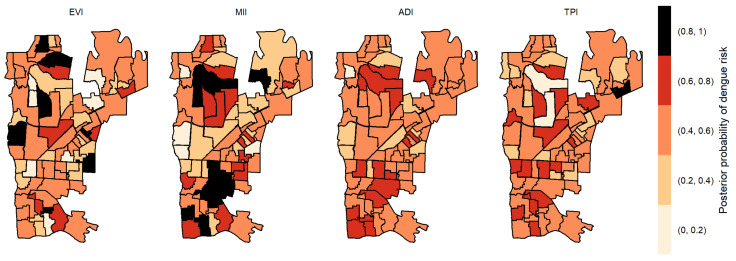
Posterior probability of dengue risk associated to infestation indexes (MII, ADI and TPI), and positive arboviral test (EVI) without time lag.

We calculated the posterior probabilities of dengue risk associated to the four indicators, TPI, ADI, MII and EVI, per each area in Foz do Iguaçu. Posterior probabilities between 0.8 and 1 suggest a high dengue risk at the specific area associated with one variable. Figure 3 illustrates the posterior probabilities by each variable without time lag, where seven non-bordered areas obtained posterior probabilities above 0.8 to EVI, 11 areas at north and south regions obtained posterior probabilities above 0.8 to MII, one area obtained a posterior probability above 0.8 to TPI, and no area obtained a posterior probability above 0.8 to ADI. The area 006 (located in the Southern part of the city) was the only one that obtained a posterior probability above 0.8 to two variables: ADI and EVI.

**Figure 4:**
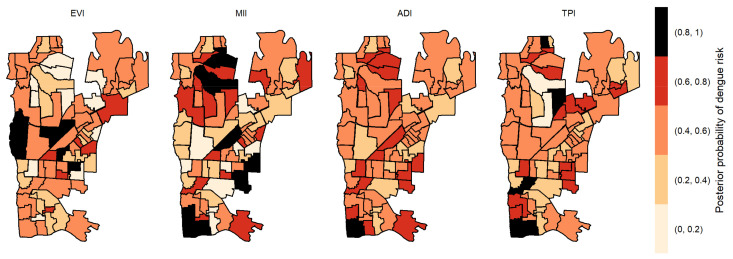
Posterior probability of dengue risk associated to infestation indexes (MII, ADI and TPI), and positive arboviral test (EVI) with one-week lag.

**Figure 5:**
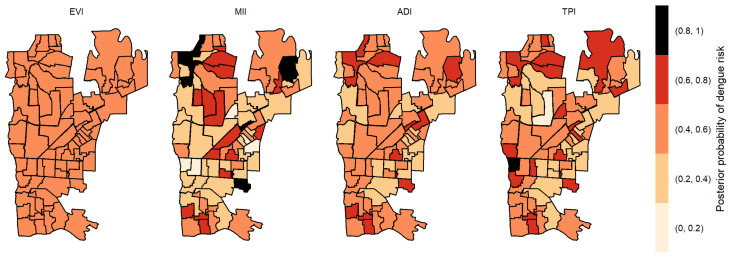
Posterior probability of dengue risk associated to infestation indexes (MII, ADI and TPI), and positive arboviral test (EVI) with two-week lag.

**Figure 6:**
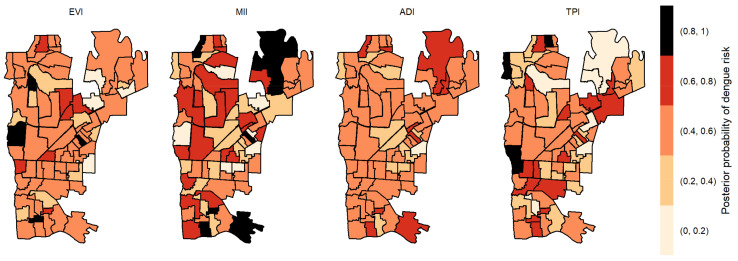
Posterior probability of dengue risk associated to infestation indexes (MII, ADI and TPI), and positive arboviral test (EVI) with three-week lag.

**Figure 7:**
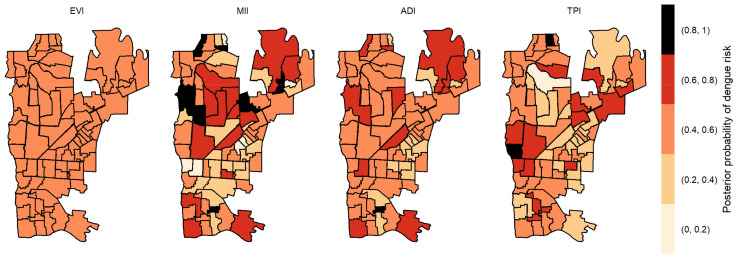
Posterior probability of dengue risk associated to infestation indexes (MII, ADI and TPI), and positive arboviral test (EVI) with four-week lag.

The posterior probabilities by each variable with one-week lag showed that six areas in the center region obtained a posterior probability above 0.8 to EVI (Figure 4). As we allowed the time lag between the notification of dengue incidence and the indicators, the number of areas with a posterior probability above 0.8 decreased. With two-week lag, only five dispersed areas obtained a posterior probability above 0.8 to MII, one to TPI and none to ADI and EVI (Figure 5). With three-week lag, seven areas obtained posterior probabilities above 0.8 to IMI, four to EVI and another four to TPI and none to ADI (Figure 6). Finally, at four-week lag, six areas obtained posterior probabilities above 0.8 to MII, two for TPI, one for ADI and none to EVI (Figure 7).

The estimation of parameters, however, carries uncertainties from the random process. Therefore, we also evaluated the 95% credibility intervals given by the posterior distribution of the random spatial effect $\beta_{ki}$. Areas with positive values for both lower and upper bounds exhibited a significant effect. Conversely, negative values for both bounds were also significant, however with a decreasing effect. We found only eight areas with significant intervals for this effect associated with EVI (Table 1), five of them with positive effects. For MII, only three areas were significant.

## 4. Discussion

Detecting the distribution and patterns of infectious diseases determines hotspot occurrence and associates them with biotic and abiotic variables is a critical step to design a more effective response [24]. The transmission of arboviruses, such as dengue, Zika and chikungunya, occurs mostly in urbanized areas in tropical regions. Cluster identification helps public health professionals detect high-risk dengue areas to prioritize for prevention and control programs [25,26]. Herein, we reported the probability of dengue risk associated with entomological indicators based on adult sampling and observed a dynamic pattern in the four weeks of evaluation but, mostly important, a low if any spatiotemporal correlation of *Ae. aegypti* density and dengue incidence. The temporal and independent spatial effects already capture a significant portion of the signal given by the human cases of arbovirus in Foz do Iguaçu, Brazil. The small number of instances of significant estimated effects due to indexes as explanatory variables confirms that only part of the process is explained by the variations in indexes. In particular, from all indexes, MII and EVI likely concentrated the remaining variation in order to evaluate number of dengue cases in the response. Considering the five time intervals and the 73 areas, there was an association between dengue risk and the entomological indicator in 12.3% of areas.

Several approaches have been used to stratify the spatiotemporal risk of arbovirus transmission. One hurdle faced by medical entomologists is to select the entomological parameter to represent mosquito infestation [18,19,20,21]. Traditionally, indexes based on larval sampling such as house (HI) and Breteau index (BI) have been the most widely used [27]. Their principle is to monitor the progress of vector eradication efforts and to protect *Ae. aegypti*-free zones from reinfestation [28]. Since larval-based indexes do not account for container productivity, HI and BI seem to have virtually no correspondence with dengue transmission [13,21,29,30]. As an alternative to larval indexes, pupal indexes have been developed to reflect the risk for transmission more meaningfully, as absolute counts of *Ae. aegypti* pupae are feasible, and the number of pupae/person is positively correlated with the number of adults mosquitoes/person [29,31]. However, in some metropolitan regions, local health agents might not be able to cover all territory due to urban violence, and indexes are highly dependent on both agent’s effort and householder availability [20,21]. Therefore, one should expect that traps are promising alternatives to larval surveys: they transfer the searching effort from the health agents to the mosquitoes themselves (this time saved allows more frequent surveys), and traps provide qualitative (% of positive traps) and quantitative (number of captures per trap) indexes [21,32]. Among several adult mosquito traps designed for capturing *Ae. aegypti*, each one with their pros and cons, the Adultrap is the one used in Foz do Iguaçu [22,23,33]. The most important drawback faced by this strategy is that detection of potentially infected mosquitoes is limited to the six times per year each trap is inspected. The Municipality of Foz do Iguaçu has to manage a balance between the number of traps in the city (3476), the personnel availability for trap sampling (80–100 health agents) and resources for molecular screening and follow the guidelines of the Brazilian Ministry of Health. Therefore, we acknowledge that, taken together, it will affect the periodicity by which traps are visited and also the likelihood of capturing a naturally infected specimen.

The use of ~3500 Adultraps installed over the city from January 2017 allowed the development of three entomological indicators directly related with trapping *Ae. aegypti* females: TPI, ADI and MII [23,33]. A fourth indicator is generated by screening for arboviruses those *Ae. aegypti* mosquitoes trapped alive: EVI. Indexes based on adult sampling had a forecasting ability for dengue occurrence in the city, especially with index estimation after 4 weeks of mosquito collection [23]. Ideally, local health managers and decision-makers would benefit if they were able, not only to determine when an outbreak is expected, but also in which parts of the city. This is particularly important in arboviruses endemic regions in which the number of health agents is insufficient to promote regular visits to local premises fortnightly. However, our efforts to correlate dengue transmission with locally derived entomological indexes pointed to an absence of powerful correlation between them.

The city of Foz do Iguaçu is divided into 76 areas, and 73 were used in this report because the other three are mainly rural areas with no traps and dengue notifications. Of them, only five areas have positive correlation between the entomological indicator and dengue incidence. Table 1 calls for attention to three different aspects of our data. First, only 9 out of 73 areas had correlation between dengue incidence and at least one entomological indicator. The TPI and ADI have no correlation with dengue incidence in the 73 areas tested using dengue incidence data from the same and four weeks of lag. This data set totals 73 × 5 (area × week lag). The rationale to elaborate the TPI follows the same principle of the house index; meanwhile, all the ~3500 Adultraps are installed in different houses. The ADI follows the rationale of the Breteau index and tries to incorporate a proxy of density of mosquitoes trapped. Both TPI and ADI do not correlate with dengue incidence, as much as HI and BI often present no match as well [23]. Therefore, our data suggests that the formulation of new indexes must still be encouraged.

Second, from the nine areas in which a correlation between dengue incidence and entomological indicators were observed, four of them a negative correlation and five a positive correlation. The positive correlation points that higher dengue incidence was observed in areas with higher MII and EVI. A positive correlation between dengue incidence and MII was expected, as much as dengue transmission is highly associated with the surroundings of human dwellings [34]. Thus, in areas with higher human population, one should expect a more intense transmission than in those areas with scattered population. However, even a lower correlation between dengue incidence and EVI brings the importance of entomo-virological surveillance as part of an early-warning system for dengue [23,35,36,37,38,39,40], Zika [41,42,43] and chikungunya viruses [43,44]. In fact, by adding entomo-virologic surveillance into their routine, health managers ideally would be able to identify hotspots of disease transmission and intensify vector control in those regions before human cases arise [35,36,42]. Therefore, the feasibility of screening field-caught *Ae. aegypti* for arboviruses and how such information can be useful for local public health managers needs to be further explored, not only in the Foz do Iguaçu context, but preferably in other endemic settings as well.

Finally, despite the fact our main objective was to evaluate whether our entomological indicators correlate with dengue incidence, a correlation between dengue incidence was only observed in some areas. For instance, investigating the variation of biotic and abiotic factors between areas with or without correlation shall provide interesting insights into local dengue epidemiology. Additional clues on local patterns of arbovirus transmission could benefit from the adoption of remote sensing techniques for mapping habitat suitability, and investigating urban landscapes could provide valuable information on differences between areas historically pointed to as hotspots and those with low entomological and/or epidemiological interest (cold spots). For instance, remotely sensed temperature data and land-cover classification were able to identify features associated with adult female *Ae. Aegypti* in an urban district in Brazil [45]. Vegetated areas and pavements were negatively associated with *Ae. Aegypti,* whereas areas with a higher percentage cover of asbestos roofs and exposed soil were positively associated with *Ae. aegypti*. Likewise, the presence and abundance of *Ae. aegypti* is multi-fold and the factors shaping mosquito distribution should be fully explored to promote an optimized vector control approach. Identifying *Ae. aegypti* hotspots and predicting those areas with higher disease transmission risk is crucial for the design of customized and optimized vector control strategies.

## Figures and Tables

**Table 1:** Areas that obtained both positive or both negative results for the 2.5/97.5% intervals in random effects variable. Other areas might include zero, which means a nonsignificant effect.

Area	EVI				MII				ADI 01234	TPI 01234
	0	1	2	3 4	01	2	3	4		
012	-									
048			-							
003	+									
039	-									
042								+		
019	+									
043					+					
046	+	+	+							
060						-				

## Data Availability

The study data is presented in the article, and further inquiries can be directed to the corresponding author. Local surveillance data requires a request to the city surveillance services.
